# Critical Current Density in *d*-Wave Hubbard Superconductors

**DOI:** 10.3390/ma15248969

**Published:** 2022-12-15

**Authors:** José Samuel Millán, Jorge Millán, Luis A. Pérez, Harold S. Ruiz

**Affiliations:** 1 Facultad de Ingeniería, Universidad Autónoma del Carmen, Cd. del Carmen C.P. 24180, Campeche, Mexico; 2 Instituto de Física, Universidad Nacional Autónoma de México, Apartado Postal 20-360, Ciudad de Mexico C.P. 04510, CDMX, Mexico; 3 School of Engineering and Space Park Leicester, University of Leicester, University Rd., Leicester LE1 7RH, UK

**Keywords:** critical current density, *d*-wave superconductors, Hubbard model

## Abstract

In this work, the Generalized Hubbard Model on a square lattice is applied to evaluate the electrical current density of high critical temperature *d*-wave superconductors with a set of Hamiltonian parameters allowing them to reach critical temperatures close to 100 K. The appropriate set of Hamiltonian parameters permits us to apply our model to real materials, finding a good quantitative fit with important macroscopic superconducting properties such as the critical superconducting temperature ($T_{c}$) and the critical current density $\left(J_{c}\right)$. We propose that much as in a dispersive medium, in which the velocity of electrons can be estimated by the gradient of the dispersion relation ∇ε(k), the electron velocity is proportional to ∇E(k) in the superconducting state (where $E\left(k\right)=\sqrt{{\left(\varepsilon \left(k\right)-\mu \right)}^{2}+\Delta^{2}\left(k\right)}$ is the dispersion relation of the quasiparticles, and ***k*** is the electron wave vector). This considers the change of ε(k) with respect to the chemical potential (μ) and the formation of pairs that gives rise to an excitation energy gap Δ(k) in the electron density of states across the Fermi level. When ε(k)=μ at the Fermi surface (FS), only the term for the energy gap remains, whose magnitude reflects the strength of the pairing interaction. Under these conditions, we have found that the *d*-wave symmetry of the pairing interaction leads to a maximum critical current density in the vicinity of the antinodal *k*-space direction (π,0) of approximately $1.407236\times 10^{8}$ A/cm^2^, with a much greater current density along the nodal direction $\left(\frac{\pi }{2},\frac{\pi }{2}\right)$ of $2.214702\times 10^{9}$ A/cm^2^. These results allow for the establishment of a maximum limit for the critical current density that could be attained by a *d*-wave superconductor.

## 1. Introduction

Despite today superconductors are known to be the keystone for overcoming the technological barriers that limit the generation of clean energy, especially at and over the MW range, for instance, in the designing of the ultra-high magnetic field coils required for fusion energy or the upgrading of >16 MW wind turbines with ultralight high temperature superconducting (HTS) generators. Surprisingly, little is known about the microscopic origin of its main physical quantity for practical applications, i.e., its critical current density, $J_{c}$. For decades, the BCS theory [1] has been the starting point to analyze the microscopic origins of most of the physical properties that appear with the transition from the normal (metallic) state to the superconducting state, giving sufficiently accurate predictions to transition parameters such as the critical temperature $T_{c}$, the critical magnetic field $H_{c1}$ (predicting the occurrence of the Meissner effect), and the temperature dependence of the energy gap Δ, which defines the amount of energy necessary to break a pair of bounded electrons (Cooper pairs) that form the superconducting state. Most of the superconducting metallic elements and alloys [2] (and all of them with critical temperatures lower than 30 K) are known to follow the BCS theory where the electron-phonon interaction is responsible for the electron pairing. It is worth mentioning that there are superconductors, with critical temperatures larger than 30 K, where the pairing is also based on the electron-phonon interaction such as MgB_2_ [3] and the copper-free oxide superconductors of the family BKBO [4]. However, there are exemptions such as the Bi and Li [5] and, more exceptionally, most practical superconducting compounds within the families of cuprates [6,7], iron pnictides, and chalcogenides [8], where the BCS coupling of electrons by the weak attraction caused by phonons does not explain all the above phenomena. Therefore, although the BCS theory explains how the density of states is changed on entering the superconducting state, which for electronically isotropic materials means that there are no electronic states anymore at the Fermi level, in the case of anisotropic electronic materials such as the LSCO, BSCCO, and YBCO cuprates [9], it is necessary to move beyond the BCS theory.

The discovery of superconductors with high critical temperatures by Bednorz and Müller [10], generally behaving as Type-II superconductors, i.e., exhibiting a mixed state where any magnetic field greater than $H_{c1}$ but lower than $H_{c2}$ (the superconducting to normal transition field) can penetrate the material in the form of vortices (fluxons), came to be the beginning of anisotropic superconductivity. This means that these materials do not have a constant or isotropic superconducting gap [6,11], such as in the BCS theory, but it depends on the wave vector (k) of the electrons that form the Cooper pairs. Therefore, although physical properties such as the critical current density in the superconductor state have been studied in the context of anisotropy, these studies mostly rely on the arguments of well-known mesoscopic or macroscopic approaches, with no consensus at the microscopic level. On the one hand, with mesoscopic approaches, we refer to fundamental approaches based on the laurate Ginzburg Landau theory, which, in brief [12], gives an account of the vortex dynamics in type II superconductors at a scale comparable to the superconducting coherence length ξ, i.e., comparable to the radius of each one of the fluxons. On the other hand, at lengths much greater than ξ, with macroscopic approaches, we mean those based on the ansatz of Bean’s model [13], where the current density J is simply constrained by a threshold value or critical current density $J_{c}$. This simple but elegant ansatz might be the first time that $J_{c}$ has been formally established as a universal parameter characterizing the practical functioning of type-II superconductors, serving as the base for building more general formulations of what today is called the critical state theory and related E–J power law approaches [14,15,16], where Maxwell equations for the superconducting state can be solved for large-scale applications such as motors, generators, fault current limiters, etc. [17,18,19,20,21].

On the other hand, with respect to microscopic theories that are beyond the conventional electron-phonon interaction, the formalism of the Hubbard model stands out [22]. This model has been successfully used to describe itinerant magnetism and the metal-insulator transition [23,24]. Such a model also allows us to consider strong correlations between electrons at different sites of the cuprate lattice, which in a periodic potential and at sufficiently low temperatures might lead to the formation of Cooper pairs. Hence, besides the phonon interaction, probably the most well-known or widely accepted electronic correlation is the long-range antiferromagnetic interaction caused by spin fluctuations [25], which is believed to give rise to the pairing between electrons because the superconductivity appears close to an antiferromagnetic state [26]. Recent studies indicate that the kinetic exchange in conjunction with the strongly correlated motion (hopping) of the carriers could be the origin of both antiferromagnetism and superconductivity in cuprates [27]. Nevertheless, as the BCS theory and the electron-phonon interaction mechanism, these interactions may not capture all the physics of all the superconductor compounds, such as the single spin and spin-triplet superconductors out of the family of cuprates [28,29]. For example, for the superconductivity in strontium ruthenate ($Sr_{2}RuO_{4}$), spin fluctuations are not enough to generate the expected superconducting triplet state and additional considerations seem necessary [30]. In this sense, the generalized Hubbard model presented here is aimed to be considered only for cuprate superconductors. This model includes first- and second-neighbor correlated hoppings, and it has been shown that the latter is a key participant in the formation of a *d*-wave superconducting gap, despite its small strength in comparison with other terms of the model. Moreover, this interaction favors the superconducting state over the phase separation, which is an important obstacle when the *d*-wave superconducting state originates from an attractive nearest-neighbor density-density interaction [31].

Thus, although at this instance we are somehow limited to considering cuprates, it is worth mentioning that these materials are also the ones of most practical interest. This is because cuprate superconductors such as YBCO and GdBCO are the key component of what is now known as the second generation of high-temperature superconducting (2G-HTS) tapes, which are being introduced into the commercial market by companies such SuperPower Inc. (East Glenville, NY, USA), American Superconductor, SuNAM, Shanghai Superconductor Technology, Theva, and SuperOx [32,33,34]. However, despite the tremendous progress in the large-scale manufacturing of superconducting tapes with fully texturized and characterized layers in continuous production capabilities, and with $J_{c}$ as their flagship parameter, its intrinsic relationship with microscopical theories is nearly unexplored. It is commonly assumed either as (i) a purely macroscopic characterization value that is measured by recording the voltage in a four-probe configuration, with the critical current $I_{c}$ defined by the condition that the voltage reaches some threshold value for the electric field at self-field conditions, typically 1 μV/cm or (ii) a physical parameter that, from the mesoscopic point of view, relates to the physics of vortices, where $J_{c}$ relates to the equilibrium between magnetic pressure and the pinning forces. Nevertheless, in these approaches, it is impossible to infer what electronically correlated mechanism gives rise to this phenomenon because even though the mesoscopic approach may consider the vortex-vortex and vortex-defects interactions as the origin of $J_{c}$, their correlations are beyond the scale of ξ. However, in the microscopic world, the first or lowest threshold for $J_{c}$ must come at lengths lower than ξ, where the Cooper pairs break for the vortex core in the mixed state. Thus, even for perfectly magnetically isotropic type-II superconductors with no defects, a $J_{c}$ must be established. This means that a closer look at our microscopic understanding of $J_{c}$ for superconducting cuprates is still needed, which will be the focus of discussion in Section 2 of this paper. Therein, the physical principles that sustain our generalized Hubbard model for *d*-wave superconductors are explained, such that the current density on the Fermi surface of cuprate superconductors such as YBCO can be calculated. Then, the dynamics of the current density along the reciprocal space and as a function of temperature are presented in Section 3, followed by a brief account of the main conclusions of this study in Section 4.

## 2. The Hubbard Model Approach and Related Considerations

Let us start by reminding the reader that to describe the dynamics of the carriers on the CuO_2_ planes of the superconducting cuprates, the formalism of the three-band Hubbard model is well-known [35]. Under this framework, the electronic states are close to the Fermi energy ($E_{F}$) and can be described reasonably well by a single-band tight-binding model on a square lattice with second-neighbor hoppings [36]. This supports the idea that, on the one hand, the current density in the superconducting state of HTS materials can be incorporated via a single-band Hubbard model on a square lattice where the second-neighbor charge-bond interaction (correlated hopping) leads to the formation of Cooper pairs with *d*-wave symmetry. On the other hand, low-temperature superconductors showing an isotropic gap in all directions can be treated by an s-wave pairing function, where a generalized Hubbard model that includes the nearest-neighbor hopping (t) and the so-called nearest-neighbor correlated hopping interaction (∆t) can explain superconducting states with an extended s-symmetry gap [37]. Thus, for *d*-wave superconductors, our model contains the nearest-neighbor (Δt) and second-nearest-neighbor ($\Delta t_{3}$) correlated hopping interactions, in addition to the on-site (U) and inter-site (V) Coulombian repulsions [38].

The mean-field electronic dispersion relation ($\varepsilon_{MF}$) in a square lattice includes the mean-field single-particle hopping that is a function of the electron density (n). Therefore, the superconducting state requires solving a couple of integral equations for a set of Hamiltonian parameters, which, in our case, is set to reach a critical temperature $T_{c}\approx 100\;K$, for the sake of generality. Additionally, within the semiclassical theory of metals, the current density (*J*) is given by J=ρqv, where ρ is the volume density of charge carriers, q is their charge, and v is their mean velocity. Notwithstanding, it is worth reminding the reader that for explaining the depth penetration phenomena in superconductors, the statements of the BCS theory imply that the mean velocity of Cooper pairs must be assumed (as a first approach) within the previous formula.

However, from the viewpoint of quantum mechanics, it is not truly possible to know whether the current density of electrons in the superconducting state satisfies a relation of the form J=ρqv, but at least some fundamental considerations can be made:

- The electrons in the superconductor state travel across the crystal at a finite velocity (v) of less than c. Otherwise, there would not be a finite critical current.
- In a dispersive medium, the velocity of electrons can be estimated by the gradient of the relation of dispersion ε(**k**), but in the case of superconductors, it needs to be estimated from the quasiparticle’s relation corresponding to Cooper pairs.
- The electronic states that mainly participate in the formation of Cooper pairs are those near the Fermi; therefore, the higher velocity corresponds to that on the FS. Thus, for a given direction of k**,** the group velocity involves the states k such that $\left|k\right|<\left|k_{F}\right|$ and $\left|v\left(k\right)\right|\leq \left|v\left(k_{F}\right)\right|$.
- The Cooper pairs are formed by electrons with the wave vectors ***k*** and −***k***, whereby an electron travels in an opposite direction from the other. Analogously to the Mott-insulator transition, hole doping is considered for the carriers’ density from when it is half-filled [39].
- In anisotropic superconductivity, the electrons with wave vectors close to the nodes have a weak superconducting gap and require very low temperatures to form the Cooper pairs; therefore, the anti-nodal states play a more dynamic role in carrying the superconducting current [40].

With the above considerations, an appropriate expression for J in a superconductor can then be written as

(1) $$J=\rho qv_{g}\;,$$

where ρ is the number of carriers per volume unit, which for YBCO tapes with J in the order of MA/cm^2^ is approximately $10^{22}/{\mathrm{cm}}^{3}$ [41], the carrier charge is q=2e, with e the bare charge of the electron. The group velocity ($v_{g}$) is given by

(2) $$v_{g}\left(k\right)=\frac{1}{\hbar }\left|\nabla E\left(k\right)\right|$$

where E(k) is the dispersion of the relation of the quasiparticles. It is important to mention that the Equation (1) J is equivalent to that of the radiant flux in one dimension ($J=\frac{1}{2m}\left(\psi^{*}p_{x}\psi +\psi p_{x}^{*}\psi^{*}\right)$, where ψ is the wave function and $p_{x}=\left(\frac{\hbar }{i}\right)\frac{d}{dx}$ is the momentum operator).

Thus, by considering a single-band generalized Hubbard model in a square lattice with first- and second neighbor correlated hoppings, Δt and $\Delta t_{3}$ respectively, together with the on-site (U) and first-neighbor (V) Coulombic repulsions, the corresponding Hamiltonian ($\hat{H}$) can be written as [38]

(3) $$\hat{H}=t\sum_{<i,j>,\sigma }c_{i\sigma }^{†}c_{j\sigma }+t^{\prime }\sum_{\ll i,j\gg ,\sigma }c_{i\sigma }^{†}c_{j\sigma }+U\sum_{i}n_{i↑}n_{i↓}+\frac{V}{2}\sum_{<i,j>}n_{i}n_{j}+\Delta t\sum_{<i,j>,\sigma }c_{i,\sigma }^{†}c_{j,\sigma }\left(n_{i,-\sigma }+n_{j,-\sigma }\right)+\Delta t_{3}\sum_{\begin{array}{c} <i,l><j,l>\\ \ll i,j\gg ,\sigma \end{array}}c_{i,\sigma }^{†}c_{j,\sigma }n_{l}\;,$$

where $n_{i}=n_{i,↑}+n_{i,↓}$, $n_{i,\sigma }=c_{i,\sigma }^{†}c_{i,\sigma }$, and $c_{i,\sigma }^{†}\;\left(c_{i,\sigma }\right)$, is the creation (annihilation) operator with spin σ=↓ or ↑ at the site i, with <i,j> and <<i,j>> denote the nearest-neighbor and next-nearest neighbor sites, respectively. The expressions for single-particle and electron-electron interaction parameters can be written in terms of the Wannier functions [$\varphi \left(r-R_{i}\right)$] centered at the lattice site $R_{i}$. Such expressions are summarized in Table 1.

Thus, after the Fourier transformation, the $c_{k,\sigma }^{†}=\frac{1}{N_{s}}\sum_{j}\exp\left(ik\cdot R_{j}\right)c_{j,\sigma }^{†}$ Equation (3) becomes

(4) $$\hat{H}=\sum_{k,\sigma }\varepsilon \left(k\right)c_{k,\sigma }^{†}c_{k,\sigma }+\frac{1}{N_{s}}\sum_{k,k^{\prime },q,\sigma }V_{k,k^{\prime },q}c_{k+q,↑}^{†}c_{-k+q,↓}^{†}c_{-k^{\prime }+q,↓}c_{k^{\prime }+q,↑},$$

where ε(k) is the single-electron dispersion relation and $N_{s}$ is the total number of lattice sites. The interaction potential ($V_{k,k^{\prime },q}$) only considers Cooper pairs with antiparallel spins, and it can be written as

(5) $$V_{k,k^{\prime },q}=U+V\beta \left(k-k^{\prime }\right)+\Delta t\left[\beta \left(k+q\right)+\beta \left(-k+q\right)+\beta \left(k^{\prime }+q\right)+\beta \left(-k^{\prime }+q\right)\right]+\Delta t_{3}\left[\gamma \left(k+q,k^{\prime }+q\right)+\gamma \left(-k+q,-k^{\prime }+q\right)\right],$$

with

(6) $$\beta \left(k\right)=2\left[\cos\left(a\;k_{x}\right)+\cos\left(a\;k_{y}\right)\right],$$

and

(7) $$\gamma \left(k.k^{\prime }\right)=4\cos\left(a\;k_{x}\right)\cos\left(a\;k_{y}^{\prime }\right)+4\cos\left(a\;k_{x}^{\prime }\right)\cos\left(a\;k_{y}\right),$$

where for the sake of generality, the effective lattice parameter a has been assumed to be equal to 3.855 Å, which corresponds to a relative average between the CuO_2_ lattice parameters found for several cuprates [42,43].

Then, the chemical potential (μ) and superconducting gap [Δ(k)] can be obtained from the mean-field BCS coupled integral equations for *d*-wave superconductors [38]:

(8) $$1=-\frac{\left(V-4{\Delta t}_{3}\right)a^{2}}{4\pi^{2}}\iint_{1BZ}\left\{\frac{{\left[\cos\left(a\;k_{x}\right)-\cos\left(a\;k_{y}\right)\right]}^{2}}{2E\left(k\right)}\tanh\left(\frac{E\left(k\right)}{2k_{B}T}\right)\right\}dk_{x}dk_{y},$$

(9) $$n-1=-\frac{a^{2}}{4\pi^{2}}\iint_{1BZ}\frac{\varepsilon \left(k\right)-\mu }{E\left(k\right)}\tanh\left(\frac{E\left(k\right)}{2k_{B}T}\right)dk_{x}dk_{y},$$

where 1BZ refers to the first Brillouin zone, which is defined as $\left[\frac{-\pi }{a},\;\frac{\pi }{a}\right]⨂\left[\frac{-\pi }{a},\;\frac{\pi }{a}\right]$. The quasiparticle energy [E(k)] is given by [44]:

(10) $$E\left(k\right)=\sqrt{{\left(\varepsilon_{MF}\left(k\right)-\mu \right)}^{2}+\Delta^{2}\left(k\right)}.$$

Therein, the mean-field dispersion relation is given by [38]

(11) $$\varepsilon_{MF}\left(k\right)=E_{MF}+t_{MF}\left[\cos\left(a\;k_{x}\right)+\cos\left(a\;k_{y}\right)\right]+4{t^{\prime }}_{MF}\cos\left(a\;k_{x}\right)\cos\left(a\;k_{y}\right),$$

with

(12) $$E_{MF}=\left(\frac{U}{2}+4V\right)n,$$

(13) $${t^{\prime }}_{MF}=t^{\prime }+2n\Delta t_{3},$$

and

(14) $$t_{MF}=t+n\Delta t,$$

where n is the number of electrons per lattice site.

Therefore, the *d*-wave superconducting gap symmetry is given by the following:

(15) $$\Delta \left(k\right)=\Delta_{d}\left[\cos\left(a\;k_{x}\right)-\cos\left(a\;k_{y}\right)\right].$$

where $\Delta_{d}$ is the temperature-dependent gap amplitude, the critical temperature of the superconductor can be determined from Equation (8) by $\Delta_{d}\left(T=T_{c}\right)=0$. On the other hand, as established by Equation (2), to calculate the superconducting critical current density (from microscopic principles), it is necessary to obtain the group velocity evaluated on the Fermi surface.

## 3. Critical Current Density

To obtain the critical current density, i.e., the maximum current density, $v_{g}\left(k\right)$ was evaluated at the Fermi surface, given by the condition $\varepsilon_{MF}\left(k\right)=\mu$, which leads to

(16) $$v_{g}\left(k_{F}\right)=\frac{a\Delta_{d}}{\hbar }\sqrt{\sin^{2}\left(a\;k_{xF}\right)+\sin^{2}\left(a\;k_{yF}\right)},$$

where a is the lattice parameter and $\Delta_{d}$ is the gap amplitude, which depends on the temperature. Therefore, the final expression for the current density is

(17) $$J\left(k_{F}\right)=\frac{1}{\hbar }\rho qa\Delta_{d}\sqrt{\sin^{2}\left(a\;k_{xF}\right)+\sin^{2}\left(a\;k_{yF}\right)},$$

where the J strength basically depends on $\Delta_{d}\left(T\right)$ for T between zero and $T_{c}$, i.e., the critical current density has the same temperature dependence as the superconducting gap amplitude. Moreover, the square root term will be the maximum for $\left(ak_{xF},a\;k_{yF}\right)\approx \left(\frac{\pi }{2},\frac{\pi }{2}\right)$, considering that the FS does not necessarily touch the point $\left(\frac{\pi }{2},\frac{\pi }{2}\right)$.

By fixing the electronic correlation and looking for the minimal energy of the ground state in the space ($t^{\prime },\;n_{op}$) with $T_{c}\approx 100$ K, we assumed that $\Delta t_{3}=0.05$ eV and Δt=0.5 eV for the case of YBCO as previously established in Ref. [45]. Likewise, for the corresponding carrier density at the Fermi surface, we assumed $\rho =1.0\times 10^{22}$ carriers/cm^3^, this is in good agreement with the recent observations reported in Ref. [41].

**Figure 1:**
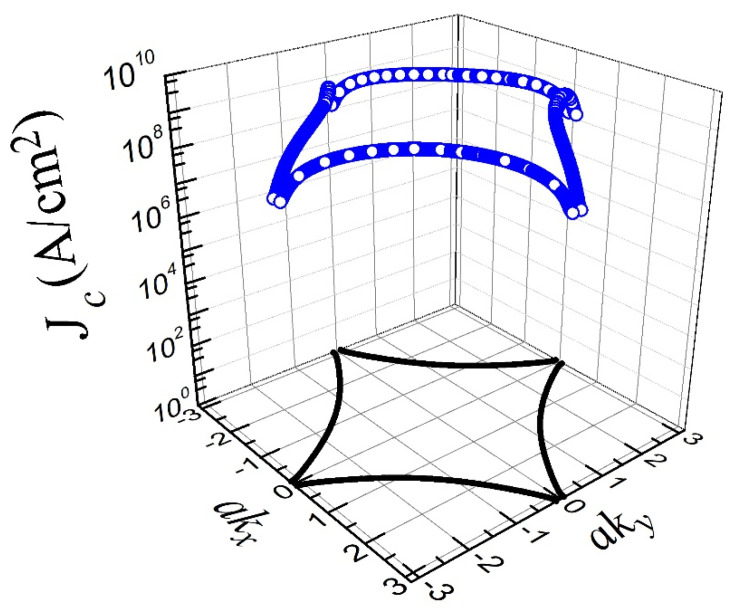
Critical current density ($J_{c}$) (blue line) as a function of ***k*** on the Fermi surface for T=0 K for a set of Hamiltonian parameters with $-t^{\prime }/t=0.06$, Δt=0.5 eV, $\Delta t_{3}=0.05$ eV, and n=0.805. The corresponding Fermi surface is depicted on the *k_x_-k_y_* plane by a black line.

In this way, by solving the above set of coupled integral equations that determine $\Delta_{d}$, the current density on the reciprocal lattice $\left(ak_{x},a\;k_{y}\right)$ of a generic YBCO crystal at the Fermi surface has been calculated (see Figure 1) for the interaction parameters already mentioned.

Figure 1 shows that the obtained FS is in good agreement with that derived from ARPES data [46] and the one obtained by similar theoretical approaches which have previously shown that the YBCO has a *d*-wave symmetry gap [47]. Therefore, as previously stated in Ref. [45], the superconducting properties of YBCO are mostly driven by the second-neighbor correlated hopping $\Delta t_{3}$, and that is not an exemption when calculating the current density of the superconductor from microscopic principles.

**Figure 2:**
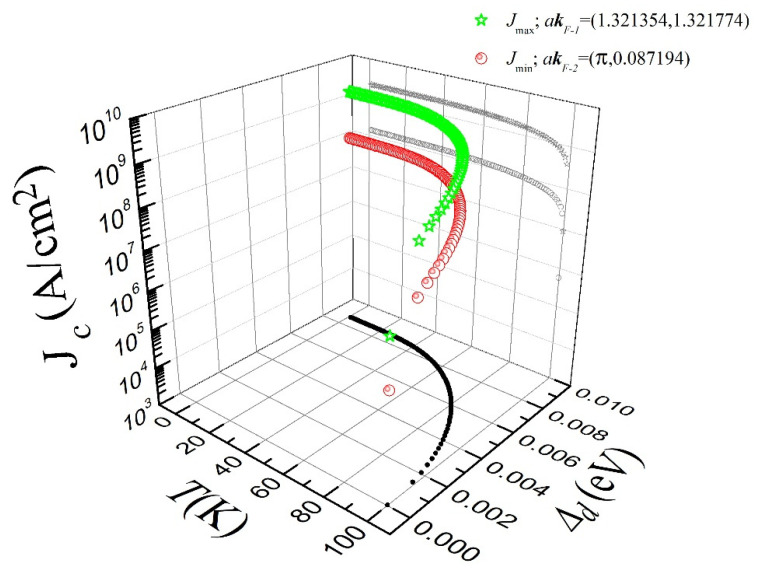
Critical current density ($J_{c}$) as a function of temperature and gap amplitude for the same system as that which Figure 1 represents. The green stars correspond to the vector ***k***_F-1_ on the FS where $J_{c}\left(k\right)$ is maximum and the red circles correspond to the vector ***k***_F-2_ where $J_{c}\left(k\right)$ is minimal. The temperature dependence of the amplitude of the *d*-wave superconducting gap ($\Delta_{d}\left(T\right)$ ) is depicted on the kx-ky plane (black solid circles).

On the other hand, when calculating the current density as a function of temperature and the gap amplitude for the same system shown in Figure 1 (but at two different **k** on the FS, say $ak_{F-1}=\left(1.321354,\;1.321774\right)$ and $ak_{F-2}=\left(\pi ,0.087194\right)$**,** represented by stars and circles in Figure 2), it is possible to notice that, as T increases, the gap amplitude $\Delta_{d}$ decreases with a very rapid drop in the current density near $T_{c}$. In fact, even for temperatures lower than that of liquid nitrogen (77 K), the microscopic current density does not change substantially (or at least not within the logarithmic scale of Figure 2). It is clear that when assuming 77 K as a reference, J slightly increases along with $\Delta_{d}$. When the temperature decreases (i.e., within the same order of magnitude), it can rapidly decrease with a parabolic tendency toward higher temperatures. This result is in good agreement with the experimental observations for $J_{c}$ along diverse YBCO samples [48,49], which means that the current density J calculated from microscopic principles is independent of macroscopic factors, such as geometry (bulk, films, crystals, etc.), as well as of mesoscopic or vortex dynamics parameters influenced by materials deposition, fabrication techniques, and composite pinning properties. J is to be understood as the most fundamental and possibly maximum critical current density that can be exhibited by the superconductor.

The above is true by reminding the well-established critical state theory for type-II superconductors [13,14,15], where it has been already proven that the current density of charge carriers into a lossless superconducting state (only possible to attain at T=0 K) is bounded by the condition $J=J_{c}$ [50]. Therefore, our model allows connecting the macroscopic world, where the critical current density characterizes the balance equation between magnetic and intrinsic pinning forces, with the microscopic world which determines the maximum current density that a superconductor can stand. However, it should be noted that due to the *d*-wave anisotropy of the superconducting gap of cuprates, J is not uniform along the Fermi surface.

**Figure 3:**
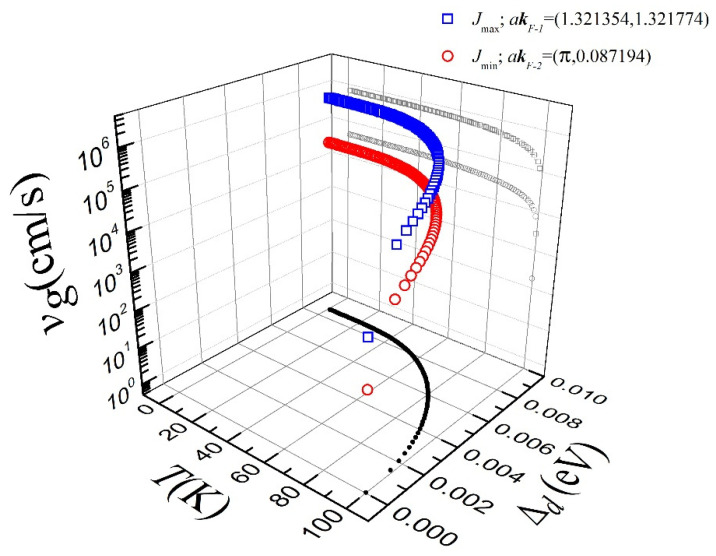
Group velocity ($v_{g}$) versus temperature and superconducting gap amplitude, evaluated at the points $ak_{F-1}=\left(1.321354,\;1.321774\right)$ (blue open squares) and $ak_{F-2}=\left(\pi ,\;0.087194\right)$ (red open circles) of the FS shown in Figure 2. The temperature dependence of the amplitude of the *d*-wave superconducting gap ($\Delta_{d}\left(T\right)$) is depicted on the horizontal plane (black solid circles).

In fact, by using high-resolution angle-resolved photoemission spectroscopy, ARPES [6,9,25], it has been demonstrated that the quasiparticle energy dispersion at the so-called nodal $\left(\frac{\pi }{2},\frac{\pi }{2}\right)$ and antinodal (π,0) directions differ. In this sense, we have found that for the calculated group velocity at T=0 K (see Figure 3), either for directions close to the node ($v_{g}=6.920943\times 10^{5}$ cm/s at $k_{F-1}$) or close to the antinode ($v_{g}=4.397612\times 10^{4}$ cm/s at $k_{F-2}$), upper and bottom limits of the current density J over the FS can be established. In particular, we have found that close to the nodal direction, the current density at $k_{F-1}$ is of about $2.214702\times 10^{9}$ A/cm^2^ for the analyzed case, while for wave vectors close to the antinodal direction, it is $J\approx 1.407236\times 10^{8}$ A/cm^2^ (see Figure 2). This result indicates the existence of a maximum threshold value for $J_{c}$ in *d*-wave superconductors of about 140 MA/cm^2^ because even though a higher current density can exist along the nodal direction due to its augmented group velocity, in practical applications, a transport current density higher than this value would destroy the superconductivity along $k_{F-2}$, creating an instantaneous avalanche of vortices moving from the superconducting state to the normal one. Thus, putting our findings into the context of the most recent measurements of the critical current density in PLD-deposited YBCO thin films [34,41], it should be noticed that, indeed, *d*-wave superconductors such as YBCO can reach ultra-high critical current densities of approximately 90 MA/cm^2^ for oxygen overdoped samples, denoting significant room for improvement. Therefore, by having shown that from microscopic principles the maximum current density that a cuprate superconductor may stand is around 140 MA/cm^2^, we reaffirm the hypothesis at Ref. [41], which suggests that overdoping strategies with oxygen post-processing treatments and nanoengineering pinning can truly offer powerful prospects to increase the limits of dissipation-free current transport in cuprate superconductors and coated conductors for practical applications. Nevertheless, regardless of how the $J_{c}$ properties of the superconductor are enhanced, either by the inclusion of pinning elements (defects, dopants, inclusions, etc.) or the increment of charge carriers by the hole doping of the superconducting CuO_2_ planes, the $J_{c}$ of the cuprates cannot exceed the microscopic limits imposed by the Cooper pairs formation.

## 4. Conclusions

In summary, in this paper, a comprehensive analysis of the critical current density for *d*-wave superconductors has been performed by using a generalized Hubbard model, which includes a second neighbor correlated-hopping term that is key for the formation. The superconducting properties were calculated by solving two coupled integral equations, Equations (8) and (9), obtained by applying the BCS formalism to the mean-field generalized Hubbard Hamiltonian, where the integrals involved can be efficiently calculated by isolating the region around the Fermi surface. Thus, the calculation of the group velocity on the FS allows estimating the maximum critical current density of quasiparticles in the superconducting state, which in turn permits the establishment of a more fundamental threshold for the critical current density $J_{c}$ (fundamental in the sense that it is related to the microscopic pairing mechanism).

Likewise, it is suggested that the k-states at the anti-nodal region play a more important role than those around the nodes in the formation of the superconducting condensate. This is because even though the nodal direction shows the highest current density, in the order of the London penetration depth (λ≈100 nm), i.e., in the length scale of fluxons for type-II superconductors, Cooper pairs must coexist at both the nodal and the antinodal regions of the CuO_2_ planes (otherwise a stable flux pinning event cannot occur). Thus, our results support the idea that the further nanoengineering of superconducting thin films based on the deposition of rare-earth barium copper oxygen (REBCO) compounds and the increment of the charge density by the p-doping of superconducting CuO_2_ planes are both likely to lead to critical current densities even higher than the record of $90\;{\mathrm{MA}/\mathrm{cm}}^{2}$ in overdoped YBCO films. Nevertheless, although these are already ultrahigh critical current densities, the capacity of the superconductors for transporting current is not unlimited, but on the contrary, from purely microscopic principles, we predicted that critical current densities higher than $140\;{\mathrm{MA}/\mathrm{cm}}^{2}$ in *d*-wave superconductors are unlikely to appear. Still, to fully ratify this conclusion, further research for the 3D case of tetragonal lattices is needed, such that families of superconductors with more Cooper oxygen planes and higher critical temperatures could be studied.

## Figures and Tables

**Table 1:** Expressions for the Hubbard model parameters with u(r) as the lattice periodic potential and $v\left(r-r^{\prime }\right)$ as the interaction potential between two electrons in the lattice.

Single-particle parameters
$t_{i,j}=\int d^{3}r\varphi^{*}\left(r-R_{i}\right)|-\frac{\hbar^{2}\nabla^{2}}{2m}+u\left(r\right)|\varphi \left(r-R_{j}\right)$
$t=t_{i,j}$ with <i,j> $t^{\prime }=t_{i,j}$ with ≪i,j≫
Electron-electron interaction parameters
$U_{ij}^{kl}=\int d^{3}rd^{3}r^{\prime }\varphi^{*}\left(r-R_{i}\right)\varphi^{*}\left(r^{\prime }-R_{j}\right)v\left(r-r^{\prime }\right)\varphi \left(r-R_{k}\right)\varphi \left(r^{\prime }-R_{l}\right)$ $U=U_{ii}^{ii}$ $V=U_{ij}^{ij}$ $U_{ii}^{ij}=\Delta t$, with <i,j>
$U_{il}^{lj}=\Delta t_{3}$, with <i,l>, <j,l>, and ≪i,j≫

## Data Availability

The data presented in this study are available on reasonable request from the corresponding author.
